# Exploring the use of narrative-based approaches in individuals with amyotrophic lateral sclerosis: A narrative review

**DOI:** 10.1017/S1478951526101965

**Published:** 2026-03-05

**Authors:** Kristie Calix, Kannaley

**Affiliations:** Palliative Care, University of Maryland Baltimore Graduate Studies in Palliative Care, Baltimore, MD, USA

**Keywords:** Amyotrophic lateral sclerosis, Lou Gehrig’s disease, motor neuron disease, narrative medicine, narrative-based intervention

## Abstract

**Objectives:**

Narrative-based approaches have been utilized in medicine to better understand the illness experiences of individuals living with chronic conditions. In particular, people with amyotrophic lateral sclerosis (pALS) may benefit from use of narrative-based approaches, given the potential impact of progressive decline on identity of self. This review explores the use of narrative-based approaches in studies involving pALS to provide further insight to the experiences and psychosocial needs of this population.

**Methods:**

A search was conducted utilizing EMBASE, CINAHL, PsycInfo, and Google Scholar with several terms related to amyotrophic lateral sclerosis (ALS) and narrative-based approaches. Studies were included if they were written in English, incorporated methods that promoted the production of narratives, and reported data that could be clearly isolated to pALS.

**Results:**

The search revealed a total of 154 articles for title and abstract screening. Fifty-two articles were selected for full-text review. Thirty-two articles met the criteria for data extraction. Four descriptive categories emerged upon examination of the narrative-based approaches implemented across the studies: psychosocial intervention, illness experience, intervention targeting specific needs, and secondary analysis of data. Some of the common themes identified across studies included: loss of physical and communicative function, adaptation to life changes, shifts in identity, and tension with the healthcare system.

**Significance of results:**

Despite the communication challenges that often coincide with disease progression, narrative-based approaches can be utilized in pALS. These approaches should be implemented to gain insight on the disease experiences of pALS, providing opportunity for patient-centered interventions to address the psychosocial needs of this population.

## Introduction

Interactions among patients and medical professionals are rooted in story, as discourse related to illness experience is nuanced by the individual’s history, beliefs, and perceptions. The value of these stories has been cultivated through the development of narrative medicine, a subfield that utilizes story as a driver for research, education, and clinical care (Kirmayer et al. 2023). Narrative medicine has evolved into a field of study and incorporates elements of provider education and competency, addresses direct clinical practice, and examines the theoretical notion of the use of narrative in medical settings (Charon 2001; Palla et al. 2024). More generally, narrative-based approaches utilize techniques that are rooted in narrative, such as open-ended interviewing or the use of psychosocial interventions that inspire the production of stories. These stories provide insight into the patient experience, allowing for the contextualization and presentation of the illness trajectory (Kirmayer et al. 2023).

The use of narrative-based approaches in medical settings provides benefits to both clinicians and patients. Narratives reveal the intricacies of the illness experience (Charon 2007), probing the, ‘narrativity of disease, of health, of healing, and of the relation between the sick person and the one who tries to help’ (Charon 2008, 26). Narrative-based approaches provide opportunities for the patient to reframe their illness, promoting a sense of healing and validation (Kirmayer et al. 2023). In turn, narrative-based approaches encourage maintenance of perceived identity through the production of story, especially within the context of changing functions and roles as part of the chronic illness experience (Clandinin et al. 2010; McLean and Pasupathi 2010; Morris 2008; Slocum 2021; Slocum et al. 2017).

Other benefits of narrative-based approaches include promotion of trust between the patient and provider (Charon 2001; Thoele et al. 2020), inspiration of provider curiosity, empathy, and understanding (Charon 2007; Charon et al. 2016; Fox et al. 2023; Kemper et al. 2020; Mehl-Madrona et al. 2021; Mirsa-Hebert et al. 2012), and reduction of compassion fatigue and burn-out due to secondary trauma (Kemper et al. 2020; Loy and Kowalsky 2024; Winkel 2016). While much of the theoretical focus of narrative medicine has emphasized the role of the provider, narrative-based approaches are more directly focused on patient responses (Gibson 2021; Kaye et al. 2022). Patient-specific benefits, such as improved quality of life (QoL) (Kheirbek et al. 2019) and sense of peace (Wise et al. 2018), have been documented in literature utilizing narrative-based approaches. Patients also report that narrative-based interventions inspire a sense of healing (Charon 2006, 2009; Charon et al. 2016; De Bemedetto et al. 2007; Divinsky 2007; Greenhalgh and Hurwitz 1998; Kalitzkus and Matthiessen 2009; Launer 2002; Lewis 2011).

While the benefits of narrative-based approaches have been documented in patients with chronic illnesses, such as cancer (Slocum et al. 2017; Wise et al. 2018) and heart disease (Kheirbek et al. 2019; Slocum et al. 2019), the use of these techniques with persons with amyotrophic lateral sclerosis (pALS) has not been reviewed. PALS may be particularly situated to benefit from narrative-based interventions due to the impact of the disease on expressive communication. ALS is a neurodegenerative disease that results in the death of motor neurons, leading to muscle atrophy and the eventual loss of controlled movements. The disease impacts the ability to independently breathe, speak, and swallow effectively over time (National Institute of Neurological Disorders and Stroke (NINDS) 2024). Individuals with locked-in syndrome (a phenomenon sometimes experienced in the final stages of ALS) are left with significantly limited communication abilities, which are sometimes confined to vertical eye movement (Laureys et al. 2005; Rowland and Schneider 2001). Previous research has emphasized the importance of effective communication with patients with locked-in syndrome, noting the alignment between effective communication and the patient’s sense of personhood (Nizzi et al. 2018; Séguin et al. 2016).

Despite expressive communication challenges, narrative-based approaches may be beneficial for gathering more information about the experiences of pALS. Narrative-based interventions may also serve as appropriate methods for therapeutic intervention to address the psychosocial complexities of the disease. The scope of this review includes: studies on interventions intended to directly benefit pALS, studies that examine the illness experience of pALS, and studies involving secondary analysis of illness narratives of pALS with the aim of providing a comprehensive review of the use of narrative-based approaches in thispopulation.

## Methods

A literature search was conducted utilizing terms related to the diagnosis of ALS and the use of narrative-based approaches. Searches were performed via the EMBASE, CINAHL, and PsycInfo databases, utilizing the terms, ‘amyotrophic lateral sclerosis’ OR ‘Lou Gehrig’s disease’ OR ‘motor neuron disease’ AND ‘narrative medicine’ OR ‘life review’ OR ‘dignity therapy’ OR ‘narrative-based intervention’ OR ‘narrative intervention’ OR ‘narrative therapy’ OR ‘narrative interview’ OR ‘storytelling’ OR ‘illness narrative’ OR ‘illness experience.’ Additionally, the same terms were utilized to gather results from the first 2 pages of Google Scholar (for each secondary term) as part of the screening process. No constrictions were placed on year of publication. Each title and abstract was uploaded into Covidence and screened using inclusion and exclusion criteria (see Table 1). A full-text article review was conducted for articles that met the inclusion criteria.

**Table 1.** Inclusion and exclusion criteria

Include	Exclude
Written in English	Written in a language other than English
The central focus, intervention, or technique utilized to gather data for the study involves a narrative-based approach	The central focus, intervention, or technique utilized to gather data for the study does not involve a narrative-based approach
Results can be isolated to the pALS/care partner population	Results cannot be isolated to the pALS/care partner population
Qualitative or quantitative studies that contain patient/care partner data	Feasibility studies (without patient data), conference abstracts/papers, editorials, full books, book chapters, and graduate thesis papers Experiences solely focused on care partners without the inclusion of PALS

## Results

**Figure 1.**
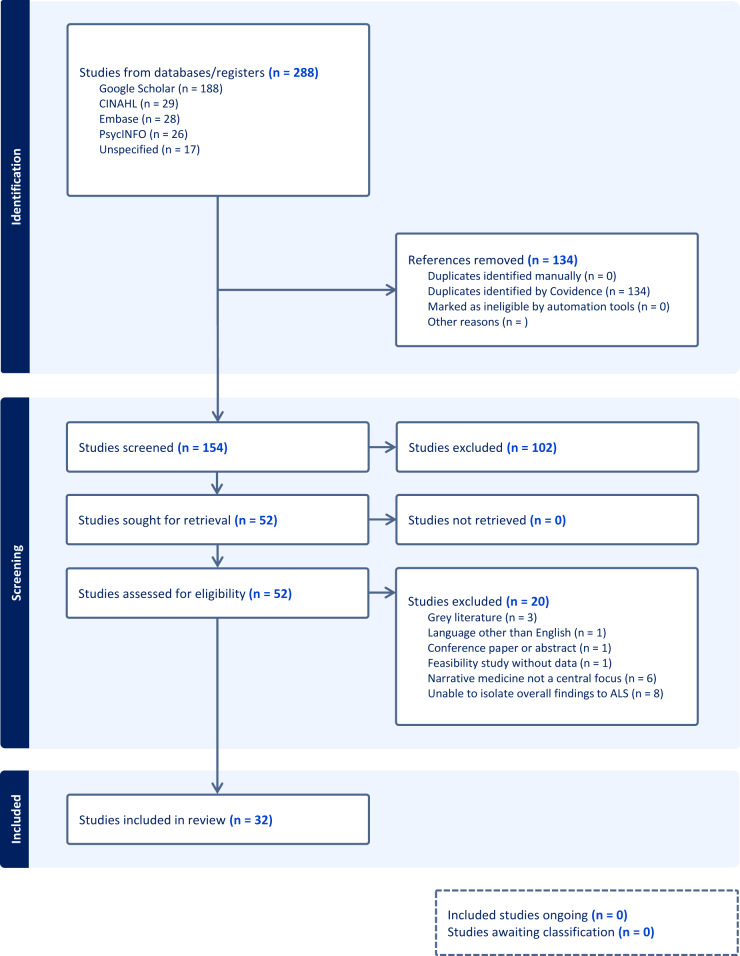
PRISMA diagram of study selection process.

Following the removal of 134 duplicate articles, 154 articles were identified for screening. One hundred two articles were excluded during the screening process, leaving a total of 52 articles for full-text review. Thirty-two studies were selected for data extraction. Figure 1 provides the PRISMA flow diagram for visualization of the study selection process.

Further analysis revealed 4 overarching classifications of articles (see Table 2). The first category, the implementation of narrative-based psychosocial intervention (*n* = 3), solely includes studies using dignity therapy with pALS. The second category (*n* = 14) features the use of narrative-based interviewing to explore the experiences of pALS. The third category (*n* = 4) consists of studies utilizing narrative-based approaches to gather data to address a particular need in the pALS population, such as exploring the reasons for lack of engagement in nationally funded social services. The final category (*n* = 7) includes articles that utilize secondary analysis of narrative data to establish overarching themes related to living with ALS.

**Table 2.** Classification of articles

**Category**	Studies	
**Narrative-based approaches for psychosocial intervention (*n* = 3)**	Aoun et al. 2015; Bentley et al. 2014; Meira et al. 2024	
**Narrative-based approaches to explore the illness experience (*n* = 16)**	*Time-focused studies*	Mistry and Simpson 2013; O’Brien et al. 2011a; Remm et al. 2019; Spoden et al. 2024; Whitehead et al. 2011
	*Sociocultural and relational studies*	Bolmsjö and Hermeren 2001; Cobb and Hamera 1986; Foley et al. 2016; Oh et al. 2014; Yuan et al. 2021
	*Psychosocial studies*	Brown and Addington-Hall 2008; Hecht et al. 2002; Locock et al. 2009; Pinto et al. 2021
	*Open-ended exploratory studies*	Gunton et al. 2021; King et al. 2009
**Narrative-based approaches to address a specific need of pALS (*n* = 4)**	Ciotti et al. 2018; Hsieh et al. 2016; Jeppesen et al. 2015; O’Brien et al. 2011b	
**Secondary analysis of narrative data**	Locock et al. 2012; Mazanderani et al. 2012; O’Brien and Clark 2006; Sakellariou 2015; Sakellariou 2016; Sakellariou et al. 2013; Sakellariou et al. 2021	

## Narrative-based approaches for psychosocial intervention: dignity therapy

Few studies (*n* = 3) explored narrative-based psychosocial intervention in pALS through the use of dignity therapy (Aoun et al. 2015; Bentley et al. 2014; Meira et al. 2024). Dignity therapy is an intervention in which individuals nearing end-of-life are presented with guiding questions to promote reminiscence of monumental life events (Chochinov 2002; Martinez et al. 2017). This intervention facilitates the exploration of advice, words of wisdom, or memories that the person nearing death wishes to highlight for their loved ones. Interviews are recorded, transcribed, revised, and returned to the participant to share as a legacy document (Chochinov 2002).

The 3 identified studies reported mixed results on the effectiveness of dignity therapy in pALS. Earlier studies (Aoun et al. 2015; Bentley et al. 2014) showed no significant differences between pre- and post-intervention quantitative measurements utilizing the Patient Dignity Inventory (PDI). On the other hand, a more recent study completed by Meira et al. (2024) found significant pre- and post-test changes in PDI scores for this population, with an even greater positive change noted in the post-intervention assessment completed a week later. Additionally, Bentley et al. (2014) found no significant differences in Herth Hope Index scores on the group level but did find statistically significant positive changes on the individual level. There were no significant differences in terms of spiritual well-being. Aoun et al. (2015) also found no significant changes in measures of patient hopefulness or anxiety and depression levels. On the other hand, Meira et al. (2024) reported significant positive effects on several physical and psychosocialmeasures.

While there are inconsistencies in the quantitative evidence for the effectiveness of dignity therapy in pALS, participants reported positive effects across all 3 studies. In general, pALS found dignity therapy to be helpful for promoting a sense of intimacy with loved ones (Aoun et al. 2015; Bentley et al. 2014) and for fostering recognition of important life goals and memories (Meira et al. 2024). PALS were generally satisfied with the intervention (Aoun et al. 2015) and found it to be acceptable (Aoun et al. 2015; Bentley et al. 2014). Dignity Therapy can promote a reduction in perceived sense of suffering (Meira et al. 2024), and many of the participants reported they would recommend the intervention to other pALS (Aoun et al. 2015; Bentley et al. 2014).

## Narrative-based approaches to explore the illness experience

Sixteen studies utilized narrative-based interviews to explore the experiences of pALS or pALS and their care partners. These papers are further organized into 4 areas of focus: experiences across different periods of the disease trajectory (*n* = 5), sociocultural aspects of living with the disease (*n* = 5), psychosocial challenges (*n* = 4), and open-ended exploratory studies (*n* = 2).

### Time-focused studies

Time-focused studies examined the illness experiences of pALS or pALS and their care partners across particular stages of the disease, such as the diagnostic period or the end stages (Mistry and Simpson 2013; O’Brien et al. 2011a; Remm et al. 2019; Spoden et al. 2024; Whitehead et al. 2011). While these studies addressed factors relevant to other subcategories described, such as the psychosocial challenges of navigating life with ALS, these studies were structured with emphasis on disease stage, making time a primary theme for the organization of each article.

Three studies (Mistry and Simpson 2013; O’Brien et al. 2011a; Remm et al. 2019) explored the ALS diagnostic experience and the transition to living with the disease through use of semi-structured or narrative interviews with either pALS or pALS and their care partners. These studies utilized thematic analysis to contextualize experiences across the populations during this stage of the disease course. Remm et al. (2019) explored logistic and psychosocial aspects of the diagnostic experience, including barriers impacting the timeliness of diagnosis, perceptions of being dehumanized by healthcare professionals, support received from loved ones, and the experience of finding a new sense of control after diagnosis. O’Brien et al. (2011a) interviewed pALS and care partners, likewise exploring psychosocial aspects of care. This article is structured by time period within the diagnostic course, beginning with the initial onset of symptoms, moving to the experience of receiving the diagnosis, then exploring the ways in which participants processed the diagnosis after confirmation. Lastly, Mistry and Simpson (2013) conducted interviews with pALS to examine the process of diagnosis followed by the transition to life after diagnosis. This study utilized a combination of thematic analysis and temporal structure to organize themes across the interviews, using direct quotes from pALS to divide each subsection of the results.

Only 2 studies (Spoden et al. 2024; Whitehead et al. 2011) focused on the end-stage and bereavement phases of the ALS journey. Whitehead et al. (2011) utilized narrative interviews with pALS and care partners, focusing on the wishes of pALS at the end-of-life and the emotional responses of care partners after the deaths of their loved ones. Similarly, Spoden et al. (2024) explored perspectives on end-of-life decision-making, particularly noting strategies used to approach these decisions. Analysis of participant interviews revealed the 2 overarching themes of avoiding thoughts and discussions related to end-of-life and taking initiative to plan for end-of-life, with some participants alternating between these strategies (Spoden et al. 2024).

### Sociocultural and relational studies

Two studies (Oh et al. 2014; Yuan et al. 2021) explored the experiences of pALS and care partners through an ethnographic lens. Oh et al. (2014) utilized several methods, including observation, photo-elicitation, and semi-structured interviewing, to collect data on pALS and care partners living in South Korea. Results were organized using the metaphor of a ‘journey,’ dividing subthemes into ‘off the course’ to describe early symptoms, ‘drifting’ to describe relational difficulties, and ‘on a new boat’ to represent shifts in goals and relationships (Oh et al. 2014, p. E6–E7). Yuan et al. (2021) utilized a phenomenological approach with semi-structured interviews to examine the experiences of Chinese pALS. This study revealed themes such as adapting sense of self, providing support for the care partner, and coming to terms with the reality of the diagnosis (Yuan et al. 2021).

Additionally, this review included 2 studies that analyzed the illness experiences of pALS with a specific focus on changes in relationships (Cobb and Hamera 1986; Foley et al. 2016). Cobb and Hamera (1986) utilized open-ended interviews with a longitudinal design to capture changes in the perspectives of pALS in regards to their relationships with loved ones and with the healthcare system. Although the sample size was limited (2 participants), this study engaged perspectives of pALS across a range of topics over the span of a year. In particular, this study highlighted changes that occurred socially with decline in expressive communication (Cobb and Hamera 1986).

Foley et al. (2016) explored the perspectives of pALS, focusing on the perceived impact of the disease on their respective care partners. Participants expressed concern about being a burden to their loved ones but also noted ways in which they provided emotional support to their families. At times, they felt obligated to align care decisions with the wishes of their care partners. They often expressed heightened concern about the well-being of their care partners and other loved ones (Foley et al. 2016).

Lastly, a single study (Bolmsjö and Hermeren 2001) utilized interviews with pALS and care partners to compare the needs and perceptions of these 2 groups. Bolmsjö and Hermeren (2001) focused on the differences between the groups, noting that they are often combined, as if the patient and the care partner were a single entity. The care partner is almost considered as an ‘appendage’ to the patient (Spackman 1991). This study brings attention to the differing experiences of pALS and care partners in terms of how they contextualize the diagnosis, how they view the future, and the types of support they feel are needed (Bolmsjö and Hermeren 2001).

### Psychosocial studies

Four studies focused on psychosocial aspects of living with ALS, primarily related to disease challenges and supportive mechanisms (Brown and Addington-Hall 2008; Hecht et al. 2002; Locock et al. 2009; Pinto et al. 2021). Two of these studies highlighted the implementation and effectiveness of coping mechanisms used by pALS (Brown and Addington-Hall 2008; Hecht et al. 2002). Brown and Addington-Hall (2008) identified 4 types of narratives used to describe the disease experience and coping mechanisms: ‘sustaining’ (focusing on the positive aspects of life), ‘enduring’ (experiencing suffering), ‘preserving’ (maintaining life), and ‘fracturing’ (losing function and having concern for the future)(204).

Additionally, Hecht et al. (2002) explored the perceptions of pALS on the most negative aspects of living with the disease. Participants commonly noted difficulties with expressive communication, social isolation, and lack of independence with self-care tasks. Results showed that loss of speech, loss of mobility, and having knowledge of the poor prognosis of the disease were the most challenging aspects of living with ALS. The most frequently identified coping mechanisms included the utilization of family support, the application of technical aids, and use of ‘unspecific coping mechanisms’ (Hecht et al. 2002, 27).

Pinto et al. (2021) utilized semi-structured interviews with pALS and care partners to examine the emotional impacts of ALS. The authors attempted to interview care partners separately from pALS but conducted some joint interviews to promote ease of communication and comfort for the participants. Following an inductive analysis, the most commonly identified triggers of emotional distress included loss of function and perceived control, uncertainty about the future, management of frequent change, and lack of support from healthcare professionals or programs. Strategies that mediated the impact of the disease included maintaining a positive outlook, finding a sense of control, receiving support from others, and taking space from the disease (Pinto et al. 2021).

Lastly, Locock et al. (2009) utilized narrative interview methods with pALS and individuals with experience caring for someone with ALS to examine biological disruption and repair, concepts that have been used to describe adaptation of the sense of self in the setting of chronic disease (Bury 1982; Charmaz 1987; Lawton 2003; Pierret 2003; Williams 2000). The authors found overarching themes related to shifts in personal identity, disruption of previous routines, changes in relationships, and exploration of strategies to adapt to change (Locock et al. 2009).

### Open-ended exploratory studies

The last subcategory of approaches included 2 studies that were open-ended in nature (Gunton et al. 2021; King et al. 2009). Participants were either engaged in questioning or encouraged to contribute narrative data through different mediums (such as through use of photos) without being prompted with a particular guiding focus. Gunton et al. (2021) utilized photovoice (Catalani and Minkler 2010) as a method to explore the experiences of pALS and their care partners. Participants were asked to take pictures that captured life with the disease. These images and captions were utilized to prompt further discussion. The study revealed themes of functional decline, isolation, hope, difficulty with the healthcare system, and having to face the diagnosis (Gunton et al. 2021).

King et al. (2009) utilized various mediums, such as field notes, prose, and photographs, as catalysts for discussion of the ALS disease experience. These data were interpreted using an ‘ongoing change and adaptation’ model that seeks to contextualize decision-making within the greater context of change (King et al. 2009, 747). Participants noted their perceptions of physical and emotional aspects of the disease as well as their reactions to change. Additionally, the study revealed methods of addressing and adapting to these changes, such as passively denying the change or utilizing functional techniques to increase independence (King et al. 2009).

## Narrative-based approaches to address a specific need of pALS

The third category of articles utilized narrative-based approaches to meet a specific need or answer a particular question that affects the larger pALS population (Ciotti et al. 2018; Hsieh et al. 2016; Jeppesen et al. 2015; O’Brien et al. 2011b). Each study was grounded in the common goal of acquiring more information related to a specific topic for the benefit of the pALS population as a whole, rather than solely the individuals involved in the study. For example, Hsieh et al. (2016) examined autobiographical memory retrieval for pALS and controls with a narrative-based approach. Results revealed that pALS actually verbalized more details in response to narrative story probes in comparison to the control population. Additionally, as the disease progressed, pALS reported more frequent reminiscence on past events compared to controls (Hsieh et al. 2016).

In a unique study by Jeppesen et al. (2015), the authors used interviews with pALS to produce journalistic narratives as a means of communicating illness experiences. The researchers combined patient responses with field notes taken during the interviews to craft narratives reflective of the content recorded. Participant stories captured elements of normality among the everyday lives of people affected by ALS, highlighting aspects of family life, loss of abilities due to the disease, and navigation of the medical system (Jeppesen et al. 2015).

From the rehabilitation field, Ciotti et al. (2018) utilized narrative-based techniques to establish functional goals for pALS within the context of the World Health Organization (WHO) International Classification of Functioning, Disability, and Health (ICF) framework (WHO 2001). Participants were engaged in narrative interviews to reveal areas of concern and importance to each person with ALS. These concerns were then mapped onto the ICF framework to establish patient-centered rehab goals (Ciotti et al. 2018).

Lastly, O’Brien et al. (2011b) conducted narrative interviews with pALS and their care partners to identify barriers to the acquisition and use of home-based social services in the United Kingdom. Interview analysis revealed that pALS experienced uncertainty regarding whether they qualified for services, had concerns about the quality and consistency of services, and maintained desire to avoid change and preserve autonomy (O’Brien et al. 2011b).

## Secondary analysis of narrative data

The final subcategory of articles involved the analysis of previously recorded data, including both elicited and unelicited narratives (Locock et al. 2012; Mazanderani et al. 2012; O’Brien and Clark 2006; Sakellariou 2015, 2016; Sakellariou et al. 2013; Sakellariou et al. 2021). In instances of elicited narratives, the data were collected from previous qualitative studies and were reanalyzed from a particular lens to explore a topic more closely. These studies generally focused on a small subset of participants. Subjects of analysis included negotiation of home modifications (Sakellariou 2015), navigation of shifts in prognosis (Sakellariou et al. 2021), exploration of methods for problem solving (Sakellariou 2016), and analysis of the use of metaphorical language (Locock et al. 2012). One secondary analysis (Mazanderani et al. 2012) used previous interview data from pALS and care partners to explore the differences in the perspectives of those affected by ALS versus Parkinson’s disease. Another study utilized data from previous dyad interviews to explore the use of joint interviewing to document illness experience (Sakellariou et al. 2013).

There was only a single study (O’Brien and Clark 2006) that examined unsolicited illness narratives that were published electronically. This study analyzed public websites created by pALS and primarily reported the demographic data of the authors as opposed to thematic content. O’Brien and Clark (2006) noted that more men participated in online publishing of the ALS illness experience compared to women and that unskilled labor was the most commonly identified profession of the authors.

## Discussion

This review examined the use of narrative-based approaches with pALS and care partners to gain insight into the application of these types of methods with this specific population. In examining the goals, structures, implementation, and results of included studies, the literature reveals opportunities for use of narrative-based approaches to promote improved understanding of the experiences of pALS, to generate ideas for solving problems that affect the greater pALS population, and to provide psychosocial or therapeutic support. While the studies were divided into 4 categories (approaches for psychosocial intervention, illness experiences, addressing specific needs, and secondary analysis of data), the types of narrative-based approaches utilized in the literature could be more simply placed into 2 overarching categories: studies that reveal the lived experiences of pALS and studies that provide a particular intervention to promote the well-being of pALS.

Several themes were identified upon examination of the studies that explored the lived experiences of pALS. Due to small sample sizes and the heterogeneity of illness experiences, the results of these studies cannot be strictly applied to the greater pALS population; however, it should be noted that there were recurrent themes across the literature (Table 3). A focus on adaptation to changes that occur with disease progression, both physically and psychologically, was prominent across numerous studies (Brown and Addington-Hall 2008; Ciotti et al. 2018; Cobb and Hamera 1986; Gunton et al. 2021; Hecht et al. 2002; Hughes et al. 2004; Jeppesen et al. 2015; King et al. 2009; Locock et al 2012; Locock et al. 2009; Mistry and Simpson 2013; O’Brien et al. 2011b; Oh et al. 2014; Pinto et al. 2021; Remm et al. 2019; Sakellariou 2015, 2016; Sakellariou et al. 2013; Sakellariou et al. 2021; Spoden et al. 2024; Whitehead et al. 2011; Yuan et al. 2021). PALS and care partners frequently highlighted the effects of decline in physical and communicative function, often noting the loss of mobility and the inability to articulate clearly as heightened concerns (Aoun et al. 2015; Brown and Addington-Hall 2008; Ciotti et al. 2018; Cobb and Hamera 1986; Foley et al. 2016; Gunton et al. 2021; Hecht et al. 2002; Jeppesen et al. 2015; King et al. 2009; Locock et al. 2012; Locock et al. 2009; Mazanderani et al. 2012; Meira et al. 2024; Mistry and Simpson 2013; O’Brien et al. 2011a; Oh et al. 2014; Pinto et al. 2021; Sakellariou 2015, 2016; Sakellariou et al. 2013; Sakellariou et al. 2021; Spoden et al. 2024; Whitehead et al. 2011; Yuan et al. 2021).

**Table 3.** Overarching themes

	Adaptation to change	Identity and sense of self	Decline in physical and communicative functions	Tension with healthcare settings and professionals
Aoun et al. (2015)		X	X	X
Bentley et al. (2014)		X		
Bolmsjö I and Hermeren				X
Brown and Addington-Hall (2008)	X	X	X	
Ciotti et al. (2018)	X	X	X	
Cobb and Hamera (1986)	X	X	X	X
Foley et al. (2016)		X	X	X
Gunton et al. (2021)	X	X	X	X
Hecht et al. (2002)	X	X	X	
Hsieh et al. (2016)				
Hughes et al. (2004)	X			
Jeppesen et al. (2015)	X	X	X	X
King et al. (2009)	X	X	X	
Locock et al. (2012)	X	X	X	
Locock et al. (2009)	X	X	X	
Mazanderani et al. (2012)		X	X	
Meira et al. (2024)		X	X	
Mistry and Simpson (2013)	X	X	X	
O’Brien and Clark (2006)		X		
O’Brien et al. (2011a)			X	X
O’Brien et al. (2011b)	X	X		X
Oh et al. (2014)	X	X	X	
Pinto et al. (2021)	X	X	X	X
Remm et al. (2019)	X	X		X
Sakellariou (2015)	X	X	X	X
Sakellariou (2016)	X	X	X	X
Sakellariou et al. (2013)	X	X	X	
Sakellariou et al. (2021)	X	X	X	X
Spoden et al. (2024)	X	X	X	
Whitehead et al. (2011)	X	X	X	X
Yuan et al. (2021)	X	X	X	

Additionally, numerous studies noted the examination and shifting of personal identity in pALS, with particular focus on changes in the perception of self as well as the perceived notions of others (Aoun et al. 2015; Bentley et al. 2014; Brown and Addington-Hall 2008; Ciotti et al. 2018; Cobb and Foley et al. 2016; Gunton et al. 2021; Hamera 1986; Hecht et al. 2002; Jeppesen et al. 2015; King et al. 2009; Locock et al. 2012; Locock et al. 2009; Mazanderani et al. 2012; Meira et al. 2024; Mistry and Simpson 2013; O’Brien and Clark 2006; O’Brien et al. 2011b; Oh et al. 2014; Pinto et al. 2021; Remm et al. 2019; Sakellariou et al. 2013; Sakellariou 2015, 2016; Sakellariou et al. 2021; Spoden et al. 2024; Yuan et al. 2021; Whitehead et al. 2011; Yuan et al. 2021). Some studies highlighted tensions between the values of pALS and the values of their medical professionals (Jeppesen et al. 2015) or care partners (Foley et al. 2016). Participants noted frustrations with their interactions with healthcare professionals and identified greater healthcare system needs (Bolmsjö and Hermeren 2001; Cobb and Hamera 1986; Gunton et al. 2021; Hughes et al. 2004; O’Brien et al. 2011a, 2011b; Oh et al. 2014; Pinto et al. 2021; Remm et al. 2019; Sakellariou 2015, 2016; Sakellariou et al. 2021; Whitehead et al. 2011). Future research should address the relationship between communication and identity of self in this population, possibly within the context of social interaction with loved ones and medical professionals.

The 3 studies that implemented a narrative-based approach to specifically address psychosocial challenges in pALS (Aoun et al. 2015; Bentley et al. 2014; Meira et al. 2024) each utilized dignity therapy, an intervention that has been highly studied across different populations. These studies revealed mixed quantitative measurement outcomes but overall positive effects from the anecdotal perspectives of patients. All other studies included in this review either addressed a particular question about living with ALS or aimed to explore the more general lived experience of this population. It could be argued that information gained from narrative-based approaches could be utilized to develop more targeted psychosocial interventions that are also rooted in narrative; however, this direct connection has not yet been made for pALS. This gap in knowledge calls for future research to address the ways in which the information gathered about lived experiences could be used to provide more robust psychosocial support for this population. Future studies should examine the use of narrative-based psychosocial intervention in pALS – whether newly developed or previously established – to promote more comprehensive psychosocial care for this population.

## Limitations

As previously noted, the heterogeneity of study type, participant focus, and overarching goals of the studies in this review may impact the direct implications for the greater pALS population. ALS is a relatively uncommon disease (Jones 2020), and the progression of functional decline is generally quite rapid (Ciotti et al. 2018), particularly in motor speech function for patients with the bulbar-onset variant of the disease (Eshghi et al. 2022). Time limitations could also be a factor, as utilizing alternative means of communication, such as partner-assisted scanning, is often time-consuming (Radtke et al. 2012). Lack of clarity regarding the disease etiology (CDC 2024) and the absence of a cure for the disease may also be factors limiting research on this population, especially in cultures that have the tendency to distance themselves from the death experience (Feifel 1977; Knutson 1970; Krant 1974; Krisman-Scott 2003). Further clarification regarding the difficulties related to implementation of narrative-based approaches with pALS should be prioritized in future studies.

Lastly, the open-ended search strategy lent itself to a broader means of organization and interpretation of the data. Nevertheless, the pALS population is understudied, and it was therefore considered practical to incorporate any relevant study to achieve an accurate representation of research in this arena.

## Conclusion

Narrative-based approaches can be utilized to explore the illness experiences of pALS and their care partners, providing opportunities to improve understanding of the lived experience of this population. Future research should consider the overarching themes presented in this review (adaptation to change, identity of self, decline in physical and communicative abilities, and tension among pALS and healthcare systems), as they have been identified as important areas of focus by pALS, in hopes of generating new methods of psychological support for this population.

## References

[ref1] Aoun S, Chochinov H and Kristjanson L (2015) Dignity therapy for people with motor neuron disease and their family caregivers: a feasibility study. *Journal of Palliative Medicine* 18(1), 31–37. doi: 10.1089/jpm.2014.0213 25314244 PMC4273180

[ref2] Bentley B, O’Connor M, Kane R, et al. (2014) Feasibility, acceptability, and potential effectiveness of dignity therapy for people with motor neurone disease. *PLoS ONE* 9(5), e96888. doi: 10.1371/journal.pone.0096888 24816742 PMC4016138

[ref3] Bolmsjö I and Hermeren G (2001) Interviews with patients, family, and caregivers in amyotrophic lateral sclerosis: comparing needs. *Journal of Palliative Care* 17(4), 236–240. doi:10.1177/082585970101700403 11813340

[ref4] Brown J and Addington-Hall J (2008) How people with motor neuron disease talk about living with their illness: A narrative study. *Journal of Advanced Nursing* 62(2), 200–208. doi: 10.1111/j.1365-2648.2007.04588.x 18394032

[ref5] Bury M (1982) Chronic illness as biographical disruption. *Sociology of Health and Illness* 23(3), 263–285. doi: 10.1111/1467-9566.ep11339939 10260456

[ref6] Catalani C and Minkler M (2010) Photovoice: A review of the literature in health and public health. *Health Educ Behav Off Publ Soc Public Health Educ* 37(3), 424–251. doi: 10.1177/1090198109342084 19797541

[ref7] Centers for Disease Control and Prevention (CDC) (2024) About amyotrophic lateral sclerosis (ALS). https://www.cdc.gov/als/abouttheregistrymain/about-amyotrophic-lateral-sclerosis-als.html (accessed 11 May 2025).

[ref8] Charmaz K (1987) Struggling for a self: Identity levels of the chronically ill. *Research in the Sociology of Health Care* 6, 283–321.

[ref9] Charon R (2001) Narrative medicine: A model for empathy, reflection, profession, and trust. *JAMA* 286(15), 1897–1902. doi:10.1001/jama.286.15.1897 11597295

[ref10] Charon R (2006) *Narrative Medicine: Honoring the Stories of Illness*. Oxford: Oxford University Press.

[ref11] Charon R (2007) What to do with stories: The sciences of narrative medicine. *Canadian Family Physician* 53, 1265–1267. 17872831 PMC1949238

[ref12] Charon R (2008) Where does narrative medicine come from? Drives, diseases, attention and the body. In Rudnytsky P and Charon R (eds.), *Psychoanalysis and Narrative Medicine*. Albany: State University of New York Press, pp. 23–36.

[ref13] Charon R (2009) Narrative medicine as witness for the self-telling body. *Journal of Applied Communication Research* 37(2), 118–131. doi: 10.1080/00909880902792248

[ref14] Charon R, Hermann N and Devlin M (2016) Close reading and creative writing in clinical education: Teaching attention, representation, and affiliation. *Academic Medicine* 91(3), 345–350. doi: 10.1097/ACM.0000000000000827 26200577 PMC4721945

[ref15] Chochinov H (2002) Dignity-conserving care—a new model for palliative care: Helping the patient feel valued. *JAMA* 287(17), 2253–2260. doi: 10.1001/jama.287.17.2253 11980525

[ref16] Ciotti S, Bianconi F, Saraceni V, et al. (2018) Narrative medicine in amyotrophic lateral sclerosis and a rehabilitation project based on international classification of functioning, disability and health. *American Journal of Physical Medicine & Rehabilitation* 97(11), 832–838. doi: 10.1097/PHM.0000000000000978 29870404

[ref17] Clandinin J, Cave M and Cave A (2010) Narrative reflective practice in medical education for residents: Composing shifting identities. *Advances in Medical Education and Practice* 20(2), 1–7. doi: 10.2147/AMEP.S13241 PMC3661238 23745070

[ref18] Cobb A and Hamera E (1986) Illness experience in a chronic disease—ALS. *Social Science & Medicine* 23(7), 641–650. doi:10.1016/0277-9536(86)90111-5 3775446

[ref19] De Benedetto M, de Castro A, de Carvalho E, et al. (2007) From suffering to transcendence. Narratives in palliative care. *Can Fam Physician* 53, 1277–1279. doi:10.1016/S0140-6736(98)06443-5 17872834 PMC1949241

[ref20] Divinsky M (2007) Stories for life: Introduction to narrative medicine. *Can Fam Physician* 53, 203–205. 17872627 PMC1949105

[ref21] Eshghi M, Yunusova Y, Connaghan K, et al. (2022) Rate of speech decline in individuals with amyotrophic lateral sclerosis. *Scientific Reports* 12, 1–13. doi: 10.1038/s41598-022-19651-1 36127362 PMC9489769

[ref22] Feifel H (1977) Death in contemporary America. In Scotch N (ed.), *New Meanings of Death*. New York: McGraw Hill, pp. 42–63.

[ref23] Foley G, Timonen V and Hardiman O (2016) “I hate being a burden”: The patient perspective on carer burden in amyotrophic lateral sclerosis. *Amyotrophic Lateral Sclerosis and Frontotemporal Degeneration* 17, 351–357. doi: 10.3109/21678421.2016.1143512 26857752

[ref24] Fox R, Park K, Hildebrand-Chupp R, et al. (2023) Working toward eradicating weight stigma by combating pathologization: A qualitative pilot study using direct contact and narrative medicine. *Journal of Applied Social Psychology* 53(3), 171–181. doi: 10.1542/peds.2019-1030

[ref25] Gibson L (2021) Notes on doctoring. *Harvard Magazine*, 7 December. https://www.harvardmagazine.com/2021/12/alumni-michael-stanley (accessed 23 February 2026).

[ref26] Greenhalgh T and Hurwitz B (1998) Why study narrative? In Greenhalgh T and Hurwitz B (eds.), *Narrative Based Medicine: Dialogue and Discourse in Clinical Practice*. London: BMJ Books, pp. 3–16.

[ref27] Gunton A, Hansen G and Schellenberg K (2021) Photovoice as a participatory research tool in amyotrophic lateral sclerosis. *Journal of Neuromuscular Diseases* 8(1), 91–99. doi: 10.3233/JND-200537 32986680 PMC8293638

[ref28] Hecht M, Hillemacher T, Grasel E, et al. (2002) Subjective experience and coping in ALS. *ALS and Other Motor Neuron Disorders* 3, 225–232. doi:10.1080/146608202760839009 12710513

[ref29] Hsieh S, Irish M, Foxe D, et al. (2016) My memories are important to me: Changes in autobiographical memory in amyotrophic lateral sclerosis. *Neuropsychology* 30(8), 920–930. doi: 10.1037/neu0000291 27182707

[ref30] Hughes R, Sinha A, Higginson I, et al. (2004) Living with motor neurone disease: Lives, experiences of services and suggestions for change. *Health and Social Care in the Community* 13(1), 64–74. doi:10.1111/j.1365-2524.2005.00530.x 15717908

[ref31] Jeppesen J, Rahbek J, Gredal O, et al. (2015) How narrative journalistic stories can communicate the individual’s challenges of daily living with amyotrophic lateral sclerosis. *Patient: Patient-centered Outcomes Research* 8(1), 41–49. doi: 10.1007/s40271-014-0088-6 25231830

[ref32] Jones P (2020) For your information: Epidemiology of ALS and suspected clusters. Available at https://www.als.org/navigating-als/resources/fyi-epidemiology-als-and-suspected-clusters. (accessed 11 May 2025).

[ref33] Kalitzkus V and Matthiessen P (2009) Narrative-based medicine: Potential, pitfalls, and practice. *The Permanente Journal* 13(1), 80–86. doi: 10.7812/TPP/09.996 PMC3034473 21373252

[ref34] Kaye E, Rockwell S, Lemmon M, et al. (2022) The art of saying nothing. *Pediatrics* 149(6), e2022056862. doi: 10.1542/peds.2022-056862 35641466 PMC9619411

[ref35] Kemper KJ, Schwatz A, Wilson P, et al. (2020) Burnout in pediatric residents: Three years of national survey data. *Pediatrics* 145(1), e2019030. doi: 10.1542/peds.2019-1030 31843859

[ref36] Kheirbek R, Marr B, and Herr D (2019) Listening to their heart: A randomized trial of narrative medicine. *Abstract of the American Heart Association's Scientific Sessions 2019*. Circulation 140(s1).

[ref37] King S, Duke M and O’Connor B (2009) Living with amyotrophic lateral sclerosis/motor neurone disease (ALS/MND): Decision-making about ‘ongoing change and adaptation.’ *Journal of Clinical Nursing* 18(5), 745–754. doi: 10.1111/j.1365-2702.2008.02671.x 19239541

[ref38] Kirmayer L, Gomez-Carrillo A, Sukhanova E, et al. (2023) Narrative medicine. In Mezzich J, Appleyard J, Glare P, Snaedal J and Wilson CR (eds.), *Person Centered Medicine*. Cham, Switzerland: Springer, pp. 235–255.

[ref39] Knutson A (1970) Cultural beliefs on life and death. In Scotch N (ed.), *The Dying Patient*. New York: Russell Sage Foundation, pp. 32–63.

[ref40] Krant M (1974) *Dying with Dignity*. Springfield, Ill: Charles C Thomas.

[ref41] Krisman-Scott M (2003) Origins of hospice in the United States: The care of the dying, 1945-1975. *Journal of Hospice & Palliative Nursing* 5(4), 205–210. doi:10.1097/00129191-200310000-00014

[ref42] Launer J (2002) *Narrative-based Primary Care*. London: CRC Press.

[ref43] Launer J (2013) Training in narrative-based supervision: Conversations inviting change. In Sommers L and Launer J (eds.), *Clinical Uncertainty in Primary Care: The Challenge of Collaborative Engagement*. New York: Springer, pp. 163–176.

[ref44] Laureys S, Pellas F, Van Eeckhout P, et al. (2005) The locked-in syndrome: What is it like to be conscious but paralyzed and voiceless? *Progress in Brain Research* 150, 495–511. doi: 10.1016/S0079-6123(05)50034-7 16186044

[ref45] Lawton J (2003) Lay experiences of health and illness: Past research and future agendas. *Sociology of Health and Illness* 25(3), 23–40. doi: 10.1111/1467-9566.00338 14498928

[ref46] Lewis B (2011) *Narrative Psychiatry: How Stories Can Shape Clinical Practice*. Johns Hopkins: Johns Hopkins University Press.

[ref47] Locock L, Mazanderani F and Powell J (2012) Metaphoric language and the articulation of emotions by people affected by motor neurone disease. *Chronic Illness* 8(3), 201–213. doi: 10.1177/1742395312443390 22457342

[ref48] Locock L, Ziebland S and Dumelow C (2009) Biographical disruption, abruption and repair in the context of motor neurone disease. *Sociology of Health and Illness* 31(7), 1043–1058. doi: 10.1111/j.1467-9566.2009.01176.x 19659736

[ref49] Loy M and Kowalsky R (2024) Narrative medicine: The power of shared stories to enhance inclusive clinical care, clinician well-being, and medical education *The Permanente Journal* 28(2), 93–101. doi: 10.7812/TPP/23.116 38225914 PMC11232909

[ref50] Martinez M, Arantzamendi M, Belar A, et al. (2017) ‘Dignity therapy,’ a promising intervention in palliative care: A comprehensive systematic review. *Palliative Medicine* 31(6), 492–509. doi: 10.1177/0269216316665562 27566756 PMC5405836

[ref51] Mazanderani F, Locock L and Powell J (2012) Being differently the same: The mediation of identity tensions in the sharing of illness experiences. *Social Science & Medicine* 74(4), 546–553. doi: 10.1016/j.socscimed.2011.10.036 22227237

[ref52] McLean K and Pasupathi M (eds) (2010) *Narrative Development in Adolescence: Creating the Storied Self*. Bellingham, WA: Springer. doi: 10.1007/978-0-387-89825-4

[ref53] Mehl-Madrona L, McFarlane P and Mainguy B (2021) Effects of a life story interview on the physician-patient relationship with chronic pain patients in a primary care setting. *The Journal of Alternative & Complementary Medicine* 27(8), 688–696. doi: 10.1089/acm.2020.0449 34185546

[ref54] Meira M, Silva R, Chochinov H, et al. (2024) Effects of dignity therapy on individuals with amyotrophic lateral sclerosis: Case studies. *Palliative and Supportive Care* 22(3), 517–525. doi: 10.1017/S1478951523001888 38178278

[ref55] Mirsa-Hebert A, Isaacson J, Kohn M, et al. (2012) Improving empathy of physicians through guided reflective writing. *International Journal of Medical Education* 3, 71–77. doi: 10.5116/ijme.4f7e.e332

[ref56] Mistry K and Simpson J (2013) Exploring the transitional process from receiving a diagnosis to living with motor neurone disease. *Psychology and Health* 28(8), 939–953. doi: 10.1080/08870446.2013.770513 23464923

[ref57] Morris D (2008) Narrative medicines: Challenge and resistance. *The Permanente Journal* 12(1), 88–96. doi: 10.7812/tpp/07-088 21369521 PMC3042348

[ref58] National Institute of Neurological Disorders and Stroke (NINDS) (2024) Amyotrophic lateral sclerosis (ALS). Available at https://www.ninds.nih.gov/health-information/disorders/amyotrophic-lateral-sclerosis-als#:∼:text=Amyotrophic%20lateral%20sclerosis%20(ALS)%2C,voluntary%20muscle%20movement%20and%20breathing. (Accessed 4 May 2025).

[ref59] Nizzi M, Blandin V and Demertzi A (2018) Attitudes towards personhood in the locked-in syndrome: from third- to first-person perspective and to interpersonal significance. *Neuroethics* 13(2), 193–201. doi: 10.1007/s12152-018-9375-6

[ref60] O’Brien M and Clark D (2006) Online illness narratives about living with motor neurone disease: A quantitative analysis. *British Journal of Neuroscience Nursing* 2(8), 410–414. doi:10.12968/bjnn.2006.2.8.22040

[ref61] O’Brien M, Whitehead B, Jack B, et al. (2011a) From symptom onset to a diagnosis of amyotrophic lateral sclerosis/motor neuron disease (ALS/MND): Experiences of people with ALS/MND and family carers—a qualitative study. *Amyotrophic Lateral Sclerosis* 12(2), 97–104. doi: 10.3109/17482968.2010.546414 21208037

[ref62] O’Brien M, Whitehead B, Murphy P, et al. (2011b) Social services homecare for people with motor neurone disease/amyotrophic lateral sclerosis: Why are such services used or refused? *Palliative Medicine* 26(2), 123–131. doi: 10.1177/0269216311398697 21383059

[ref63] Oh H, Schepp K and McGrath B (2014) A journey of suffering: Living with amyotrophic lateral sclerosis in South Korea. *Journal of Neuroscience Nursing* 46(3), E3–E11. doi: 10.1097/JNN.0000000000000054 24796478

[ref64] Palla I, Turchetti G and Polvani S (2024) Narrative medicine: Theory, clinical practice and education—a scoping review. *BMC Health Services Research* 24, 1116. doi: 10.1186/s12913-024-11530-x 39334149 PMC11428871

[ref65] Pierret J (2003) The illness experience: State of knowledge and perspectives for research. *Sociology of Health and Illness* 25(3), 4–22. doi: 10.1111/1467-9566.t01-1-00337 14498927

[ref66] Pinto C, Geraghty A, Yardley L, et al. (2021) Emotional distress and well-being among people with motor neurone disease (MND) and their family caregivers: A qualitative interview study. *BMJ Open* 11(8), e044724. doi: 10.1136/bmjopen-2020-044724 PMC8372816 34404695

[ref67] Radtke J, Tate J and Happ M (2012) Nurses’ perceptions of communication training in the ICU. *Intensive and Critical Care Nursing* 28(2), 16–25. doi: 10.1016/j.iccn.2011.11.005 22172745 PMC3264744

[ref68] Remm S, Halcomb E and Stephens M (2019) Experiences of being diagnosed with motor neuron disease: “I just want to know.” *Collegian* 26, 550–555. doi: 10.1016/j.colegn.2019.02.002

[ref69] Rowland L and Schneider N (2001) Amyotrophic lateral sclerosis. *The New England Journal of Medicine* 344, 1688–1700. doi: 10.1056/NEJM200105313442207 11386269

[ref70] Sakellariou D (2015) Home modifications and ways of living well. *Medical Anthropology* 34(5), 456–469. doi: 10.1080/01459740.2015.1012614 25730663

[ref71] Sakellariou D (2016) Enacting varieties of subjectivity through practices of care: A story of living with motor neuron disease. *Qualitative Health Research* 26(14), 1902–1910. doi: 10.1177/1049732315584744 25918113

[ref72] Sakellariou D, Boniface G and Brown P (2013) Using joint interviews in a narrative-based study on illness experiences. *Qualitative Health Research* 23(11), 1563–1570. doi: 10.1177/1049732313508014 24122517

[ref73] Sakellariou D, Nissen N and Warren N (2021) The lived temporalities of prognosis: Fixing and unfixing futures. *The Cambridge Journal of Anthropology* 39(2), 138–155. doi: 10.3167/cja.2021.390210

[ref74] Séguin P, Moulin A, Fornoni L, et al. (2016) Retrospective study of the acute period of locked-in syndrome: Consciousness recovery and communication restoration. *Annals of Physical and Rehabilitation Medicine* 59, e151–e152. doi: 10.1016/j.rehab.2016.07.337

[ref75] Slocum R, Hart A and Guglin M (2019) Narrative medicine applications for patient identity and quality of life in ventricular assist device (VAD) patients. *Heart & Lung: The Journal of Acute and Critical Care* 48(1), 18–21. doi: 10.1016/j.hrtlng.2018.09.013 30539722

[ref76] Slocum R, Howard T and Villano J (2017) Narrative medicine perspectives on patient identity and integrative care in neuro-oncology. *Journal of Neuro-Oncology* 134(2), 417–421. doi: 10.1007/s11060-017-2542-5 28669013

[ref77] Slocum RB (2021) Breaking the spell: Narrative medicine applications for psychogenic nonepileptic seizures (PNES). *Seizure* 86, 96–101. doi: 10.1016/j.seizure.2021.01.01 33582585

[ref78] Spackman A (1991) *The Health of Informal Carers*. Southampton: Institute of Health Policy Studies, University of Southampton.

[ref79] Spoden C, Wenzel O, Erdmann A, et al. (2024) Coping and end-of-life decision-making in ALS: A qualitative interview study. *PLOS ONE* 19(6), e0306102. doi: 10.1371/journal.pone.0306102 38924023 PMC11207121

[ref80] Thoele D, Gunalp C, Baran D, et al. (2020) Health care practitioners and families writing together: The three-minute mental makeover. *The Permanente Journal* 24(1). 19.056. doi: 10.7812/TPP/19.056 PMC6907914 31852046

[ref81] Whitehead B, O’Brien M, Jack B, et al. (2011) Experiences of dying, death and bereavement in motor neurone disease: A qualitative study. *Palliative Medicine* 26(4), 368–378. doi: 10.1177/0269216311410900 21712334

[ref82] Williams S (2000) Chronic illness as biographical disruption or biographical disruption as chronic illness? Reflections on a core concept. *Sociology of Health and Illness* 22(1), 40–67. doi: 10.1111/1467-9566.00191

[ref83] Winkel A (2016) Narrative medicine: A writing workshop curriculum for residents. *MedEdPORTAL* 12, 10493. doi: 10.15766/mep_2374-8265.10493 30984835 PMC6440423

[ref84] Wise M, Marchand L, Roberts L, et al. (2018) Suffering in advanced cancer: A randomized control trial of a narrative intervention. *Journal of Palliative Medicine* 21(2), 200–207. doi: 10.1089/jpm.2017.0007 29135330 PMC5797325

[ref85] World Health Organization (WHO) (2001) International classification of functioning, disability and health: ICF. accessed 11 May, 2025. http://www.who.int/classifications/icf/en/

[ref86] Yuan M, Peng X, Zeng T, et al. (2021) The illness experience for people with amyotrophic lateral sclerosis: A qualitative study. *Journal of Clinical Nursing* 30(9-10), 1455–1463. doi: 10.1111/jocn.15697 33559184 PMC8248064

